# Physical Activity, Sedentary Behavior, and Long‐Term Changes in Aortic Stiffness: The Whitehall II Study

**DOI:** 10.1161/JAHA.117.005974

**Published:** 2017-08-07

**Authors:** Sara Ahmadi‐Abhari, Severine Sabia, Martin J. Shipley, Mika Kivimäki, Archana Singh‐Manoux, Adam Tabak, Carmel McEniery, Ian B. Wilkinson, Eric J. Brunner

**Affiliations:** ^1^ Research Department of Epidemiology and Public Health University College London London United Kingdom; ^2^ INSERM Centre for Research in Epidemiology & Public Health Hôpital Paul Brousse Bâtiment France; ^3^ Department of Medicine Semmelweis University Faculty of Medicine Budapest Hungary; ^4^ Clinical Pharmacology Unit University of Cambridge United Kingdom

**Keywords:** aortic stiffness, physical exercise, pulse wave velocity, Aging, Exercise, Risk Factors, Cardiovascular Disease, Epidemiology

## Abstract

**Background:**

Physical activity is associated with reduced cardiovascular disease risk, mainly through effects on atherosclerosis. Aortic stiffness may be an alternative mechanism. We examined whether patterns of physical activity and sedentary behavior are associated with rate of aortic stiffening.

**Methods and Results:**

Carotid–femoral pulse wave velocity (PWV) was measured twice using applanation tonometry at mean ages 65 (in 2008/2009) and 70 (in 2012/2013) years in the Whitehall‐II study (N=5196). Physical activity was self‐reported at PWV baseline (2008/2009) and twice before (in 1997/1999 and 2002/2003). Sedentary time was defined as sitting time watching television or at work/commute. Linear mixed models adjusted for metabolic and lifestyle risk factors were used to analyze PWV change. Mean (SD) PWV (m/s) was 8.4 (2.4) at baseline and 9.2 (2.7) at follow‐up, representing a 5‐year increase of 0.76 m/s (95% CI 0.69, 0.83). A smaller 5‐year increase in PWV was observed for each additional hour/week spent in sports activity (−0.02 m/s [95% CI −0.03, −0.001]) or cycling (−0.02 m/s [−0.03, −0.008]). Walking, housework, gardening, or do‐it‐yourself activities were not significantly associated with aortic stiffening. Each additional hour/week spent sitting was associated with faster PWV progression in models adjusted for physical activity (0.007 m/s [95% CI 0.001, 0.013]). Increasing physical activity over time was associated with a smaller subsequent increase in PWV (−0.16 m/s [−0.32, −0.002]) compared with not changing activity levels.

**Conclusions:**

Higher levels of moderate‐to‐vigorous physical activity and avoidance of sedentary behavior were each associated with a slower age‐related progression of aortic stiffness independent of conventional vascular risk factors.

**Clinical Perspective**

**What Is New?**

- More time spent in physical activity, especially sports activity, was associated with slower progression of aortic stiffening over 5 years independent of conventional cardiovascular risk factors.
- More time spent sitting for leisure was associated with faster progression of aortic stiffness independent of physical activity levels.

**What Are the Clinical Implications?**

- The effects of physical activity and sedentary time on the process of vascular aging may partly explain the link between physical inactivity and cardiovascular disease risk.

## Introduction

Physical inactivity, a modifiable risk factor for cardiovascular disease,^1^, ^2^ accounted for an estimated 13.4 million disability‐adjusted life years lost, and incurred $53.8 billion in healthcare costs worldwide in 2013.^3^ Physical inactivity and sedentary behavior are linked to cardiovascular disease, primarily through its effects on atherosclerosis.^2^ Aortic stiffness may also be implicated. However, less is known about the effects of physical activity on this marker of vascular aging.

Aortic stiffness results from loss of elasticity of the thoracic aorta because of age‐related degeneration of elastin fibers in the arterial media, accumulation of collagen, and deposition of calcium.^4^, ^5^ Aortic stiffness leads to increased systolic blood pressure, high left ventricular afterload and hypertrophy, decreased diastolic blood pressure, and reduced coronary perfusion.^5^, ^6^ Noncardiac effects of aortic stiffness include transmission of pressure pulsations to smaller arterial segments and pressure overload in the microcirculation, and consequent damage to end organs such as the brain and kidneys.^5^, ^6^ Clinically, this pathology translates to functional limitation,^7^ excess cardiovascular events, and premature mortality.^8^

Arterial stiffness has been shown to be associated with low levels of physical activity^9^, ^10^, ^11^, ^12^, ^13^, ^14^, ^15^, ^16^ and sedentary behavior.^14^, ^17^, ^18^, ^19^ However, previous studies are cross‐sectional, include only 1 assessment of arterial stiffness, or are small, short‐duration trials.^20^, ^21^ Reverse causality, an inherent limitation of cross‐sectional studies, has limited the ability to draw unbiased inferences from previous reports. Longitudinal studies with repeat measurements of pulse wave velocity (PWV) on large population samples are more informative of the nature, temporality, and direction of the associations. However, longitudinal evidence is scarce, and the association of physical activity or sedentary behavior with progression of aortic stiffness over time is not known.

Carotid–femoral PWV is the “gold standard” noninvasive method to assess aortic stiffness.^22^, ^23^ In this study, we examined whether type and intensity of physical activity, and sedentary behavior were associated with progression of aortic stiffness over 5 years in a highly phenotyped community‐dwelling cohort with repeat measurements of PWV.

## Methods

### Study Sample

We used data from the ongoing Whitehall II cohort study. Briefly, 10 308 male and female London‐based civil servants, aged 35 to 55 years, were recruited to the study in 1985 (response rate was 73%).^24^ Participants have since been followed up with questionnaire surveys and clinical examinations every 4 to 5 years. Ethical approval was obtained from the NHS National Research Ethics Service and the local Research Ethics Committee. Written informed consent was obtained from all participants at each follow‐up clinic. PWV was first measured at the 2008–2009 clinical assessment, which forms the baseline for this analysis. The present study sample includes 5196 participants who had at least 1 measurement of physical activity, and PWV assessed at the 2008–2009 (n=4347) or the 2012–2013 (n=4485) clinics.

### Aortic PWV and Blood Pressure

With the participant in a supine position, blood pressure was measured twice after 10 minutes of rest. From the supine systolic blood pressure and diastolic blood pressure, mean arterial pressure in mm Hg was calculated as: diastolic blood pressure + 0.33(systolic blood pressure − diastolic blood pressure). PWV was then assessed between the carotid and femoral sites using applanation tonometry (SphygmoCor, Atcor Medical, Australia). The time difference between the peak of the R‐wave on ECG and the foot of the pulse waveform captured by the tonometer at the site of the carotid pulse determines the blood transmission time between the heart and carotid pulse. Blood transmission time between the heart and femoral pulse was measured by the same method. The transit time was defined as the difference between the heart–carotid and heart–femoral blood transmission times. Path length was determined with a tape measure by subtracting the carotid–sternal notch distance from the femoral–sternal notch distance. PWV (m/s) was calculated by dividing the path length over the transit time.^25^ In each participant, PWV was measured twice and if the difference in velocity between the 2 measurements was larger than 0.5 m/s, a third measurement was taken. The average of the measurements was used in the analysis. PWV measurements were repeated within 30 days for 125 participants at PWV baseline and 114 participants at follow‐up to assess short‐term repeatability. Median difference of the repeats were 0.83 m/s (interquartile range 0.43–1.40) at baseline and 0.89 m/s (0.41–1.47) at follow‐up.

### Assessment of Physical Activity

Physical activity was assessed using a questionnaire 3 times over the follow‐up (1997/1999, 2002/2003, 2008/2009). At each assessment, participants were asked about the length of time (hours/week) they have engaged in the following activities over the past 4 weeks: walking, sports (cycling, soccer, golf, swimming, other sports), gardening (weeding, mowing, other gardening activities), housework (carrying heavy shopping, cooking, hanging out washing, other housework), do‐it‐yourself (DIY) activity (washing a car, painting, or decorating, other DIY), and 2 open‐ended questions on other activities. This 20‐item questionnaire was a modified version of the previously validated Minnesota physical activity questionnaire.^26^, ^27^

For each activity, including open‐ended items, a metabolic equivalent (MET) value was assigned using a compendium of activity energy costs.^28^ Activities lower than 3 METs (eg, dishwashing, boating) were categorized as mild, 3 METs to 5.9 METs (eg, cycling, weeding) as moderate, and activities of 6 METs or above (eg, swimming, mowing) as vigorous physical activity.^29^ Total physical activity was estimated as MET‐hours/week, the sum of the product of the intensity (MET) and weekly duration (hours/week) of all reported activities.

A compound total physical activity level combining duration and intensity of all activities was compiled and categorized into high (≥2.5 h/week moderately energetic or ≥1 h/week vigorous physical activity), low (<1 h/week moderately energetic and <1 h/week vigorous physical activity), and medium (all other) levels of physical activity.

Information on sedentary behavior was obtained from the 1997–1999 data collection phase using 2 questions on the time spent sitting at work/commuting and the time spent sitting at home (watching television, sewing, at desk).

### Assessment of Covariates

Body mass index and waist circumference were collected using standard protocols.^24^ Serum total cholesterol was determined on a Roche P Modular platform (2008–2009) after an overnight fast or at least 4 hours after a fat‐free breakfast. Participants were asked about frequency of alcohol consumption and cigarette smoking status (categorized into current‐, ex‐, and never‐smokers) on questionnaire surveys at each phase.

Clinical history of diabetes mellitus, myocardial infarction, coronary artery disease, angina, stroke, and hypertension were ascertained by self‐reported doctor diagnosis on questionnaire surveys, or data linkage with hospital episode statistics data for hospitalization with the conditions, and verification from medical records where available. Medication use was examined by questionnaire survey and assessment of medications brought by the participant to the clinic visit.

### Statistical Analysis

The cross‐sectional association between first‐ever measurement of PWV and physical activity for each participant was examined using linear regression. Adjusted mean PWV at each level of physical activity was calculated at age 65 years. Covariates in all multivariable analyses were age, sex, ethnicity, mean arterial pressure, heart rate, body mass index, waist circumference, smoking status, alcohol intake, total cholesterol levels, hypertension medication, and history of diabetes mellitus and cardiovascular disease. Less than 5% of participants had missing values in 1 or more covariates. Missing values were replaced by the latest available observation.

Linear mixed models were fitted to estimate 5‐year change in PWV per 1‐hour/week increase in each type of physical activity (sports, cycling, walking, housework, gardening, DIY activities), by intensity of activity (mild, and moderate to vigorous activity), total activity (total MET‐hours/week), and sedentary behavior (sitting time watching television, or for work/commute). Five‐year change in PWV per 1‐hour increase in each type of activity was estimated using a fixed‐effect interaction term with follow‐up time, allowing for subject‐specific random effects for the intercept. Multivariable models were mutually adjusted for every other type of physical activity and covariates. The association of change in PWV with moderate‐to‐vigorous activity was adjusted for hours/week of mild activity and vice versa. Linear mixed models involving sedentary time were adjusted for total MET‐hours/week of physical activity and concurrently measured confounders.

Change in level of physical activity between 2003/2004 and 2008/2009 was categorized as no change (remaining in the same low, medium, or high category), increase, or decrease. Five‐year change in PWV (2008/2009 to 2012/2013) following change in level of physical activity was estimated using a fixed‐effect interaction term between categories of change in physical activity (no change [reference category], increased, decreased) and follow‐up time, allowing for subject‐specific random effects for the intercept in the linear mixed model adjusted for confounders.

All analyses were repeated stratified by sex and age group (below and over 65 years old). Since the associations were qualitatively similar in sex‐stratified analyses, all results are presented as sex‐combined. The analyses were repeated, excluding participants with a history of myocardial infarction, coronary artery disease, angina, or stroke (sensitivity analysis). Statistical significance was defined as *P*<0.05. All analyses were performed using Stata 14.2 (StataCorp., College Station, TX).

## Results

Mean (SD) age of participants at baseline of this analysis (2008/2009) was 65 (5.8) years, 73% were men, and 25% had low and 57% had high levels of physical activity. Participants included in this study who had higher levels of physical activity had lower PWV, lower body mass index and waist circumference, were more likely to consume alcohol daily, less likely to be current smokers, have a history of cardiovascular disease, diabetes mellitus, or be on antihypertensive medication (Table 1). Characteristics of study participants by tertiles of PWV are presented in Table S1.

**Table 1:** Baseline Characteristics of Whitehall II Study Participants by Level of Physical Activity (2008–2009)

	Physical Activity			*P* Value^a^
	Low	Medium	High	
Men				
N	861	627	2309	
Age (y)	65.0 (5.9)	65.4 (6.1)	65.7 (5.6)	0.006
White ethnicity (%)	785 (91.2%)	569 (90.8%)	2209 (95.7%)	<0.001
PWV, m/s	8.7 (2.0)	8.6 (2.1)	8.5 (2.0)	0.03
Systolic blood pressure, mm Hg	124.7 (15.0)	125.8 (15.4)	125.6 (14.9)	0.09
Total cholesterol, mmol/L	5.0 (1.1)	5.0 (1.0)	5.1 (1.0)	0.28
Body mass index, kg/m^2^	26.7 (4.1)	26.4 (3.8)	26.2 (3.5)	0.001
Waist circumference, cm	96.0 (11.2)	94.9 (10.4)	93.8 (9.7)	<0.001
Smoking				<0.001
Current	70 (8.2%)	42 (6.7%)	106 (4.6%)	
Former	380 (44.2%)	289 (46.2%)	1172 (50.8%)	
Never	409 (47.6%)	295 (47.1%)	1030 (44.6%)	
Daily alcohol consumption	417 (49.1%)	286 (45.9%)	1253 (54.7%)	<0.001
History of cardiovascular disease	108 (12.5%)	63 (10.1%)	251 (10.9%)	0.27
History of diabetes mellitus	34 (4.0%)	16 (2.6%)	56 (2.4%)	0.07
Antihypertensive medication	329 (38.2%)	228 (36.4%)	747 (32.4%)	0.004
Women				
N	426	295	666	
Age	65.3 (5.9)	66.0 (6.0)	65.3 (5.7)	0.22
White ethnicity (%)	345 (81.0%)	259 (87.8%)	606 (91.0%)	<0.001
PWV, m/s	8.4 (2.2)	8.4 (2.1)	8.1 (1.8)	0.007
Systolic blood pressure, mm Hg	122.1 (16.1)	122.3 (17.1)	121.6 (17.1)	0.16
Total cholesterol, mmol/L	5.4 (1.1)	5.4 (1.1)	5.6 (1.0)	0.61
Body mass index, kg/m^2^	27.3 (5.5)	26.5 (4.9)	26.4 (4.9)	0.01
Waist circumference, cm	85.9 (12.7)	84.2 (11.5)	83.3 (11.8)	0.002
Smoking				0.19
Current	17 (4.0%)	20 (6.8%)	28 (4.2%)	
Former	156 (36.7%)	106 (36.2%)	267 (40.2%)	
Never	252 (59.3%)	167 (57.0%)	370 (55.6%)	
Daily alcohol consumption	113 (27.1%)	92 (31.6%)	236 (35.8%)	0.01
History of cardiovascular disease	42 (9.9%)	22 (7.5%)	44 (6.6%)	0.14
History of diabetes mellitus	20 (4.7%)	9 (3.1%)	16 (2.4%)	0.07
Antihypertensive medication	148 (34.7%)	98 (33.2%)	193 (29.0%)	0.11

PWV indicates pulse wave velocity. Values are mean (SD) for continuous variables, or numbers (%) for categorical variables.

a *P* for linear trend.

Mean (SD) PWV (m/s) was 8.4 (2.4) and 9.2 (2.7) at baseline and follow‐up, respectively. Participants with high levels of physical activity had lower PWV (8.44 m/s [95% CI 8.37, 8.51]) at age 65 years compared with those with low levels of physical activity (8.57 [95% CI 8.47, 8.67]; *P* for trend 0.02; Table S2) in cross‐sectional analyses.

Mean (SD) 5‐year increase in PWV was 0.76 m/s (95% CI 0.69, 0.83). Total MET‐hours/week of physical activity was not associated with PWV change (Table 2). In analyses by intensity of physical activity, each additional hour/week of moderate to vigorous activity at baseline was associated with −0.02 (95% CI −0.04, −0.001) smaller increase in PWV over 5 years compared with the mean 5‐year increase of 0.76 m/s. Mild activity was not associated with PWV change. By type of physical activity, each additional hour/week engaged in sports or cycling activities were associated with −0.02 m/s (95% CI −0.03, −0.001) and −0.02 m/s (95% CI −0.03, −0.008) smaller increases in PWV over 5 years in models adjusted for other types of activity, respectively. Household activities such as gardening, housework, DIY, and walking were not associated with change in PWV.

**Table 2:** Association Between Intensity and Type of Physical Activity (2008/2009) and Sedentary Time (1997/1999) With 5‐Year Change in PWV (Between 2008/2009 and 2012/2013)

	5‐Year Change in PWV	
	Mean Difference (95% CI)^a^	
	Model 1	Model 2
Total physical activity (per 1‐MET h/w)	−0.002 (−0.004, 0.0004)	−0.001 (−0.003, 0.001)
Intensity of physical activity (per 1‐h/w)		
Moderate to vigorous^b^	−0.028 (−0.046, −0.011)	−0.018 (−0.035, −0.001)
Mild	0.006 (−0.002, 0.014)	0.006 (−0.002, 0.015)
Type of physical activity (per 1‐h/w)		
Sports^b^	−0.021 (−0.037, −0.006)	−0.016 (−0.031, −0.001)
Housework	0.006 (−0.004, 0.016)	0.007 (−0.003, 0.017)
Gardening	0.006 (−0.008, 0.021)	0.009 (−0.005, 0.023)
Do it yourself	−0.010 (−0.030, 0.011)	−0.007 (−0.027, 0.014)
Walking (per 1‐MET h/w)	0.002 (−0.001, 0.004)	0.001 (−0.002, 0.004)
Cycling (per 1‐MET h/w)^c^	−0.019 (−0.027, −0.010)	−0.016 (−0.025, −0.008)
Sedentary time in 1997/1999 (per 1‐h/w)		
Sitting for leisure^b^	0.007 (0.001, 0.013)	0.007 (0.001, 0.013)
Sitting at work/commute^c^	−0.013 (−0.017, −0.009)	−0.011 (−0.016, −0.007)

Model 1 values are adjusted for age, sex, ethnicity, and mean arterial pressure. Model 2 values are additionally adjusted for heart rate, body mass index, waist circumference, smoking, alcohol intake, total cholesterol levels, history of cardiovascular disease, diabetes mellitus, and hypertension medication. MET indicates metabolic equivalent; PWV, pulse wave velocity.

a Mean difference compared with average 5‐year increase in PWV 0.76 m/s (95% CI 0.69, 0.83).

b *P*<0.05.

c *P*<0.001.

In longitudinal analyses of change in level of physical activity (2003/2004 to 2008/2009) in relation to change in PWV (2008/2009 to 2012/2013), participants who increased their level of physical activity had −0.16 (95% CI −0.32, −0.002) smaller increases in PWV over 5 years compared with those who did not change their activity level, whereas those who decreased their level of physical activity had larger increases in PWV (Figure).

**Figure 1:**
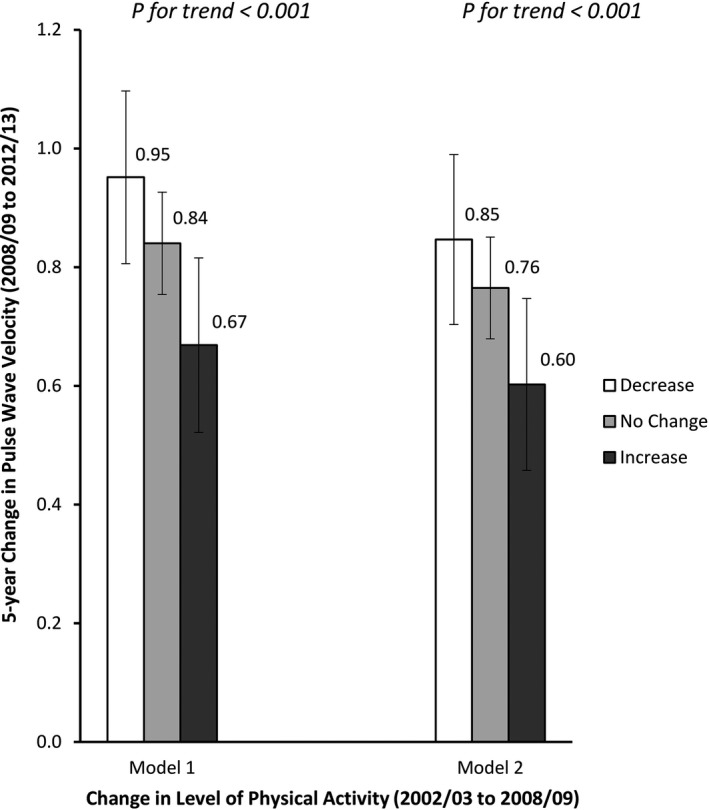
Five‐year change in pulse wave velocity (between average age 65 to 70 years) by change in level of physical activity (between average age 60 to 65 years). Model 1 values are adjusted for age, sex, ethnicity, and mean arterial pressure. Model 2 values are additionally adjusted for heart rate, body mass index, waist circumference, smoking, alcohol intake, total cholesterol levels, history of cardiovascular disease, diabetes mellitus, and hypertension medication. Error bars represent 95% CI.

Each additional hour/week leisure sitting (eg, watching television) was associated with 0.007 m/s (95% CI 0.001, 0.013) larger increase in PWV over 5 years independent of total physical activity and other confounders, whereas time sitting at work or during commute was associated with decreased elevation in PWV (Table 2).

In analyses stratified by age, magnitudes of the association were similar among younger (≤65 years) and older (65+ years) participants (*P* for interaction>0.2). All associations remained unchanged in analyses excluding participants with a history of cardiovascular disease, angina, myocardial infarction, or stroke (not shown).

## Discussion

This prospective study showed that higher levels of moderate‐to‐vigorous physical activity are associated with a slower rate of aortic stiffening. The effects were largely independent of known cardiovascular risk factors. Participants typically engaged in sports, such as cycling, swimming, or soccer, had slower aortic stiffening. However, this effect was not observed for less energetic forms of activity, such as housework, gardening, or DIY. Analysis of change in physical activity in relation to change in PWV suggested one fifth of the effect of aging on aortic stiffness may be prevented by increasing levels of physical activity. Independent of the level of physical activity, longer time watching television or spent on other sedentary leisure interests was associated with higher rates of aortic stiffening.

To our knowledge, this is the first population‐scale longitudinal study with repeated measurements of physical activity and PWV. Existing trials enrolled few participants and are short in duration,^20^, ^21^ limiting their generalizability and the ability to draw conclusive inferences from their findings. Cross‐sectional studies have shown inverse associations between physical activity and various measures of arterial stiffness among younger^17^, ^30^, ^31^, ^32^ and older adults.^9^, ^10^, ^11^, ^12^, ^13^, ^14^, ^15^, ^16^ Considering lower exercise levels could in principle be the consequence of, rather than the risk factor for, greater aortic stiffness, interpretation of the findings of cross‐sectional studies are ambiguous. Our longitudinal finding of slower PWV progression in the group that increased their physical activity over time is a novel and important mechanistic pointer. It provides good evidence that the physical activity effect is primary. Furthermore, it suggests that interventions targeting an increase in physical activity could have a sizeable effect on arterial stiffening, leading to potentially important public health outcomes.

We examined rate of progression of aortic stiffening over time in relation to different intensities and types of activity. Moderate‐to‐vigorous, but not mild, activity was associated with slower progression of aortic stiffness over time. Consistent with our finding, mild intensity activity on its own was not associated with carotid artery stiffness in young Dutch adults.^33^ Other studies suggest that mild activity such as walking confers benefit in terms of lower arterial stiffness only among older, but not middle‐aged adults.^11^, ^34^

Sports activities were particularly effective in slowing arterial aging. Holding other types of activity constant, an hour/week increase in sports activities was associated with decreased progression of PWV. This effect was not observed for housework, gardening, DIY activities, or walking. Consistently, meta‐analyses of individually underpowered experimental trials suggested that aerobic exercise, but not resistance training, was associated with decreased PWV over the intervention period.^20^, ^21^ The association of sports, but not domestic activities, with slower aortic stiffness might be explained by cardiorespiratory fitness, which was found in The Northern Ireland Young Hearts project to mediate the association between sports and arterial stiffness.^30^

To date, the biological mechanisms through which physical activity is related to aortic stiffness are not well established. Physical exercise is related to improved vascular compliance and remodeling,^35^, ^36^, ^37^ improved endothelial function because of increased laminar shear stress,^38^, ^39^ reduced production of vasoconstrictors such as endothelin I and angiotensin II,^40^ reduced oxidative stress,^40^, ^41^ and reduced low‐grade inflammation,^42^ all of which may explain the protective role of physical activity, and more specifically sports activities, for aortic stiffness.

Sedentary behavior and physical activity are separate and distinct phenomena that may coexist. Among physically active Whitehall II study participants who engaged in 3 or more hours/week of sports, 25% reported 4 or more hours a day watching television or sitting for leisure interests. With total level of physical activity held constant, we found those who spend more time watching television or engaged in other types of leisurely sedentary interests had higher rates of aortic stiffening independent of cardiovascular risk factors. This finding supports previous studies that found higher risk of diabetes mellitus, cardiovascular disease, cancer, and mortality in those with high sedentary time, independent of physical activity levels.^43^, ^44^, ^45^ In contrast, sitting time during commute or at work seemed to slow aortic stiffening over time. The difference between the effect of sitting for leisure and for work or commute can be attributable to confounding by unaccounted lifestyle factors such as snacking on high‐fat food items associated with leisure time sitting. Moreover, sitting time at work and during commute were combined in our data, whereas the latter may be more active. Breaks in sedentary sitting (such as those during commute or work) were shown to mitigate the negative effects on metabolic outcomes^46^, ^47^ and endothelial function,^48^ and thus might explain the inverse association of work/commuting time sitting and aortic stiffness. Previous cross‐sectional studies found sitting time at weekends, but not weekdays, was correlated with carotid artery stiffness among young adults^18^ and that the association of sedentary time and arterial stiffness disappeared after adjustment for number of breaks in prolonged sitting time.^19^

The strengths of this study lie in the large sample size and repeat measurements of PWV, physical activity, and potential confounders, using the same devices and standardized protocols. We minimized the possibility of reverse causation (ie, low physical activity because of stiffer arteries) by examining the association of activity and sedentary patterns with subsequent progression of aortic stiffness over time, and by excluding participants with a medical diagnosis of cardiovascular disease or stroke in sensitivity analysis.

A limitation of this study is self‐report of sedentary time and physical activity over the past 4 weeks. The error in measurement of physical activity is random and independent of PWV values, which means any resulting bias is likely to have weakened the observed associations toward the null. In assessment of PWV, the pulse wave transit time is measured with great precision using applanation tonometry. The pulse path length is measured by a measuring tape on the body surface, which although it is based on a standardized protocol, yields an approximation of the true distance. A more precise estimation of path length would require magnetic resonance imaging or application of invasive methods, which are not feasible in epidemiological studies. Our carotid–femoral PWV method based on applanation tonometry is nevertheless considered the “gold standard” for large studies,^22^, ^23^ and is an independent predictor of fatal and nonfatal cardiovascular events in various populations.^6^, ^8^, ^49^ The method is recommended for management of hypertension as an intermediate end point for cardiovascular events.^23^

In conclusion, our findings suggest that increasing sports activities and reducing television viewing and other sedentary leisure time may have beneficial vascular effects in slowing the process of aortic stiffening. This finding may partly explain the link between physical inactivity and cardiovascular disease risk.

## Sources of Funding

The Whitehall II study is supported by grants from the British Heart Foundation (RG/13/2/30098 and RG/16/11/32334), British Medical Research Council (K013351), and the US National Institute on Aging (R01AG013196 and R01AG034454). Brunner is supported by the British Heart Foundation (RG/13/2/30098 and RG/16/11/32334) and the European Commission (FP7 project no. 613598). Kivimäki is supported by a professorial fellowship from the Economic and Social Research Council, and NordForsk, the Nordic Programme on Health and Welfare. Wilkinson is a British Heart Foundation senior fellow. McEniery and Wilkinson received support from the Cambridge National Institute for Health Research Biomedical Research Centre. Funding sources did not have a role in the design and conduct of the study, the collection, management, analysis, and interpretation of the data or the preparation, review, approval, or decision to submit the manuscript.

## Disclosures

None.

## Supporting information


**Table S1.** Baseline Characteristics of Whitehall II Study Participants by Tertiles of Pulse Wave Velocity (2008–2009)
**Table S2.** Cross‐Sectional Association Between Intensity and Type of Physical Activity (2008/2009) and Sedentary Time (1997/1999) With Pulse Wave Velocity (2008/2009)
