# Supplementary material for: Physical Activity, Sedentary Behavior, and Long‐Term Changes in Aortic Stiffness: The Whitehall II Study
Source: J Am Heart Assoc. 2017 Aug 7;6(8):e005974. doi: 10.1161/JAHA.117.005974 (PMC5586440; doi:10.1161/JAHA.117.005974)
Supplement: Supplementary file 1 — Table S1. Baseline Characteristics of Whitehall II Study Participants by Tertiles of Pulse Wave Velocity (2008–2009) Table S2. Cross‐Sectional Association Between Intensity and Type of Physical Activity (2008/2009) and Sedentary Time (1997/1999) With Pulse Wave Velocity (2008/2009) [file JAH3-6-e005974-s001.pdf]

Supplemental Material

Table S1. Baseline Characteristics of Whitehall II study participants by tertiles of pulse wave velocity (2008-2009)

	Tertiles of PWV at baseline			Spearman's Rho[=]
	1	2	3	
**Pulse Wave velocity (m/s)**	6.6 (0.6)	8.1 (0.4)	10.7 (1.7)	-
**Age**	63.1 (4.9)	64.9 (5.5)	68.1 (5.6)	0.38**
**Total MET-hours/week of Physical activity**	49.9 (29.1)	48.7 (28.7)	48.6 (29.9)	-0.04*
**Intensity of Physical activity**				
**Moderate to Vigorous (hours/week)**	4.1 (3.5)	3.8 (3.5)	3.7 (3.4)	-0.06**
**Mild (hours/week)**	11.0 (7.1)	11.1 (7.2)	11.4 (8.0)	-0.004
**Type of Physical activity**				
**Sports (hours/week)**	3.0 (4.2)	2.5 (3.8)	2.4 (3.9)	-0.08**
**Housework (hours/week)**	3.0 (4.1)	3.1 (4.3)	3.1 (4.3)	-0.02
**Gardening (hours/week)**	7.2 (5.9)	7.2 (6.3)	7.3 (6.3)	-0.01
**Do It Yourself (hours/week)**	1.7 (3.1)	1.7 (3.0)	1.6 (3.0)	-0.005
**Walking (hours/week)**	7.1 (5.7)	7.2 (5.6)	7.6 (6.4)	0.01
**Cycling (hours/week)**	0.4 (1.8)	0.3 (1.4)	0.2 (1.3)	-0.07**
**Sedentary Time in 1997/99**				
**Sitting for Leisure (hours/week)**	20.2 (10.7)	21.8 (11.9)	23.0 (11.6)	0.11**
**Sitting at Work/Commute (hours/week)**	21.9 (15.9)	18.9 (15.7)	14.9 (14.9)	-0.19**
**Systolic Blood pressure (mmHg)**	117.5 (13.4)	124.0 (13.9)	132.1 (15.5)	0.42**
**Total Cholestrol (mmol/l)**	5.3 (1.0)	5.2 (1.0)	5.2 (1.1)	-0.05**
**Body Mass Index (Kg/m2)**	25.4 (3.8)	26.4 (3.8)	26.8 (3.9)	0.17**
**Waist Circumference (cm)**	88.1 (10.9)	91.9 (11.2)	94.1 (11.2)	0.23**
**Smoking**				0.008
**Current**	82 (5.7 %)	87 (6.0 %)	62 (4.3 %)	
**Former**	622 (43.0 %)	679 (47.0 %)	678 (46.9 %)	
**Never**	741 (51.3 %)	680 (47.0 %)	705 (48.8 %)	
**Daily Alcohol Consumption**	661 (46.6 %)	690 (48.6 %)	650 (45.7 %)	0.02
**History of Cardiovascular disease**	110 (7.6 %)	123 (8.5 %)	187 (12.9 %)	0.08**
**Anti-hypertensive medication**	344 (23.8 %)	474 (32.8 %)	604 (41.7 %)	0.17**

Values are mean (standard deviation) for continuous variables, or numbers (%) for categorical variables.
[=] Spearman's correlation coefficient between baseline PWV (continuous) and baseline characteristics.
* P-value < 0.05
** P-value < 0.001

Table S2. Cross-sectional association between intensity and type of physical activity (2008/09) and sedentary time (1997/99) with pulse wave velocity (2008/09).

	Mean Difference in Pulse Wave Velocity (95% Confidence Interval)[=]	
	**Model 1**	**Model 2**
**Total Physical activity level**		
Low	Ref	Ref
Medium	-0.15 (-0.31, 0.01)	-0.01 (-0.16, 0.15)
High *	-0.37 (-0.50, -0.25)	-0.13 (-0.25, -0.01)
**Total Physical activity (Per 1-Met hour/Week)**	-0.003 (-0.004, -0.0009)	0.0001 (-0.0016, 0.0019)
**Intensity of Physical activity (per 1-hour/week)**		
Moderate to Vigorous *	-0.049 (-0.065, -0.033)	-0.019 (-0.035, -0.004)
Mild	0.004 (-0.003, 0.011)	0.005 (-0.001, 0.012)
**Type of Physical activity (per 1-hour/week)**		
Sports *	-0.043 (-0.056, -0.029)	-0.020 (-0.033, -0.006)
Housework	0.006 (-0.003, 0.016)	0.005 (-0.004, 0.014)
Gardening *	-0.021 (-0.034, -0.008)	-0.014 (-0.027, -0.001)
Do It Yourself	-0.002 (-0.021, 0.016)	0.004 (-0.014, 0.022)
Walking (Per 1-MET hour/week) *	0.003 (0.000, 0.005)	0.002 (0.000, 0.005)
Cycling (Per 1-MET hour/week) **	-0.013 (-0.020, -0.005)	-0.007 (-0.014, 0.000)
**Sedentary Time in 1997/99 (Per 1-hour/week)**		
Sitting for Leisure	0.004 (-0.001, 0.009)	0.002 (-0.004, 0.008)
Sitting at Work/Commute *	-0.004 (-0.008, 0.000)	-0.004 (-0.008, 0.000)

Model 1 values are adjusted for age, sex, ethnicity, and mean arterial pressure. Model 2 values are additionally adjusted for heart rate, body mass index, waist circumference, smoking, alcohol intake, total cholesterol levels, history of cardiovascular disease, diabetes, and hypertension medication.

[=] Mean difference compared to mean baseline pulse wave velocity 8.4 (SD 2.4) m/s.

* P-value < 0.05

** P-value < 0.001
